# The neural substrate of self- and other-concerned wellbeing: An fMRI study

**DOI:** 10.1371/journal.pone.0203974

**Published:** 2019-10-01

**Authors:** HanShin Jo, Yang-Yen Ou, Chun-Chia Kung

**Affiliations:** 1 Dept. of Psychology, National Cheng Kung University (NCKU), Tainan, Taiwan; 2 Inst. Of Medical Informatics, NCKU, Tainan, Taiwan; 3 Dept. of Electrical Engineering, NCKU, Tainan, Taiwan; 4 Mind Research and Imaging (MRI) Center, Tainan, Taiwan; Southwest University, CHINA

## Abstract

Happiness, or Subjective Well-Being (SWB), is generally considered as a peaceful and satisfied state accompanied by consistent and optimistic mood. Due to its subjective and elusive nature, however, wellbeing has only been scarcely investigated in the neuroimaging literature. In this study, we investigated its neural substrates by characterizing two different perspectives: self- or other-concerned wellbeing. In the present study, 22 participants evaluated the subjective happiness (with button presses 1 to 4) to 3 categories (intra- and inter-personal and neutral) of pre-rated pictures in a slow event-related fMRI. Because wellbeing is constantly featured by pleasure feelings after self-inspection, we predict that happier conditions, featured by “intra-personal vs. neutral” and “inter-personal vs. neutral” conditions, should yield higher BOLD activities in overlapping reward- and self-related regions. Indeed, medial prefrontal (mPFC), pregenual ACC (pACC), precuneus and posterior cingulate cortex (PCC) were revealed both by General Linear Model (GLM) (categorical contrasts) and parametric modulations (correlations with rating 1-4s), specifically, more connectivity between nucleus accumbens (NAcc) and mPFC, via additional psychophysiological interaction, or PPI, analyses. More interestingly, GLM and multivariate searchlight analyses jointly reveal the subdivision of mPFC and the PCC/precuneus, with anterior mPFC and dorsal PCC/precuneus more for interpersonal, posterior mPFC and ventral PCC/precuneus more for intrapersonal, SWB, respectively. Taken together, these results are not only consistent with the “cortical midline hypothesis of the self”, but also extending the “spatial gradients of self-to-other-concerned processing” from mPFC to including both mPFC and PCC/precuneus, making them two “hubs” of self-to-other-concerned wellbeing network.

## Introduction

While there is no universal definition, the term happiness or wellbeing generally describes the feeling of positive and pleasure emotion with “contentment” [1] and “consistent, optimistic” mood [2]. Desiring happiness is an important motivation for achieving the quality of life, and has been extensively studied by philosophers about its origin [3,4]. Historically, happiness has been discussed under two schools of thoughts: hedonic and eudaimonic. The hedonic happiness is from the Greek philosopher Aristippus and later adapted by the utilitarian philosophers emphasizing basic instincts of seeking pleasure and avoiding suffering or pain [5]. The hedonic happiness are later characterized as a combination of the higher ratio of positive to negative emotion (affective) and life satisfaction (cognitive), and merged into the Subjective Well-Being (SWB) theory [6]. Another kind of happiness, eudaimonic, bears its roots from works of Aristotle, describing happiness as realizing human potentials and living lives to its fullest [5,7]. The eudaimonic happiness was integrated into the Psychological Well-Being (PWB) theory [8], featuring six different factors essential to one’s well-being: relatedness, autonomy, personal growth, self-acceptance, purpose in life, and environmental mastery [9–11].

Although the bifactor (SWB and PWB) models of wellbeing [12] are independent and measuring distinct aspects of happiness, these models overlap at general wellbeing constructs [13,14]. For example, social values, such as social interactions and relationships with others, are strong predictors for both SWB and PWB [12,15,16]. In addition, eudaimonic wellbeing are moderated by both affective (e.g., passion) and cognitive (life satisfaction) factors [17–19]. Third, Barrett-Cheetham, Williams, & Bednall [20] found that positive emotions (e.g. gratitude, compassion, pride, contentment) may differently be associated with self-/other-focused eudaimonic wellbeing. Four, the dissociation of self- and other-concerned happiness has been postulated as a theoretical model by Dambrun & Ricard [21]. Lastly, the self-centeredness, characterized by fluctuating phases of wellbeing and ill-being, is relatively focused on personal interests and egoism. In contrast, selflessness is represented by altruism, kindness, respect, empathy, and compassion, integrating the internal and external environment as a whole [22]. Collectively, these literature jointly suggest a burgeoning approach to examine distinctive mechanisms of wellbeing by self- and other-concerned dimension [23,24].

Although there were significant increases in the number of wellbeing related studies in recent years, only a handful of neuroimaging studies have directly explored the neural substrates of wellbeing. Three of studies directly explored the neural substrates of wellbeing found positive correlation between the gray matter volume in right precuneus and scores of affective and cognitive factors in SWB [25], left gray matter volume in left precuneus and eudaimonic hedonic balance (or EHB, see [26]), and bilateral insular cortex with sub-scales of eudaimonia in PWB [27]. Another two resting-state fMRI studies revealed that default mode network (DMN) may play an important role during the wellbeing, the positive correlation was found between the DMN brain areas and inclination to ruminate [28], and between DMN regions and eudaimonics wellbeing [26]. In addition, among the studies investigated happiness from affective aspects, the first [29] scanned professional actors by having them recall happy episodes, such as declaration of love, the birth of own child, or a family reunions. The authors found relative brain activations in the orbitofrontal, medial prefrontal, and ventrolateral prefrontal cortex. More recently, Rutledge, Skandali, Dayan, & Dolan [30] used the gambling paradigm to operationalize the momentary happiness/SWB as the prediction error of the money gained relative to the expected gain/loss. Although an influential study, to us such quantitative definition of happiness by monetary value is less conventional. Lastly, Kringelbach & Berridge [31–33] summarized the neuroimaging findings linking with happiness and pleasure. They found that mechanisms of pleasure ‘encoding’ similar to the encoding of ‘happiness’, emphasizing the importance of the ‘hedonic hotspots’—the reward-related brain regions like: nucleus accumbens (NAcc), ventral pallidum, brain stem, mPFC, and insular cortices. To us, these brain regions may be well related to the wellbeing processing, too.

The reasons of so few (f)MRI studies addressing wellbeing, seem to us, are likely the following: first, wellbeing is a subjective (and sometimes vague) concept; second, even people can self-evaluate wellbeing, it may also be hard to keep people engage, then disengage, and then re-engage in wellbeing in the scanner setting. These might be the reasons behind why the Rutledge, Skandali, Dayan, & Dolan [30] study adopted the unexpected monetary reward—for it is quantifiable, precise, and ideal for the parametric event-related fMRI. But still, equating the durable and positive state of wellbeing with the wellbeing from unexpected monetary reward in gambling is a bit contentious. Therefore in the present study, we adapt the parametric event-related fMRI, but ask participants directly to evaluate their wellbeing by viewing three kinds of pictures: one for inducing intrapersonal wellbeing, which is more self-concerned and achievement-oriented stimuli; another for inducing interpersonal wellbeing, which contains more self-less, and other-concerned pictures; and the third the neutral condition, such as office scenes, lone contemplation, etc. By the earlier literature review, we hypothesized that both Inter- and Intra-personal wellbeing would, relative to the neutral conditions, recruit brain regions for positive affect, rewarding, and pleasure. Moreover, subdivisions in some brain regions, especially related to self- and reward processing, including mPFC [26,34] and precuneus, have been shown to reveal spatially distinctive regions along self-other dimensions. We expect the similar spatial gradient to be similarly revealed by the intrapersonal/self-concerned and interpersonal/other-concerned wellbeing contrasts. Lastly, we will report various analysis results, including general linear model, parametric modulation, and multivariate searchlight mapping [35], to identify common and converging findings across individual analyses, to not only compare with the extant literature, but also converge our findings.

## Methods

### Participants

This study was approved by the NCKU Research Ethics Committee (REC) with the approval number 103-050-2. All participants gave their written consent before the experiment. Twenty-two healthy participants, half male, and without neurological disorders, joined the scanning (mean age = 22.3 yrs; SD = 3.5 yrs). Three participants were excluded due to excessive head movements, errors in the codes or missing responses, leaving 19 participants included in the data analyses. All participants were compensated with $500 NTD for their 1-hour participation.

### Stimuli and task

Three stimulus conditions were sampled: 150 intrapersonal happiness events, 200 interpersonal happiness events, and 200 neutral events. These 550 pictures were collected from Google images by keywords like: happiness, SWB, Xing-Fu. For intrapersonal subtype: achievement, graduation, promotion, game/sports/monetary winning, rewarding; for interpersonal subtype: family, friendship, union, parent-child, lovers; and for neutral subtype: working, thinking, speculating, idling, office, and mundane activities. The collected pictures were independently examined by a group of 35 undergrads participants, instructed to evaluate how much of the happiness feeling each picture induced in themselves, rather than evaluating how happy the people in the pictures were, on a 1 to 4-point scale, with 1 being the least happy, and 4 the most happy. To ease the burden, the number of pictures rated by each participant was 200, with 66 or 67 photos randomly drawn for each category. The average ratings of 29 successfully coded subjects were: 2.987±0.651, mean rt = 2.76s for intrapersonal happiness subtype, 3.535±0.45, mean rt = 2.86s for the interpersonal happiness subtype, and 1.732±0.674, mean rt = 2.53s for the neutral subtype. The pictures for the fMRI experiments were again randomly drawn from the same 550 photo pool, with 6 pictures per category (totally 18 trials per run) into a 10-run list per subject.

**Fig 1:**
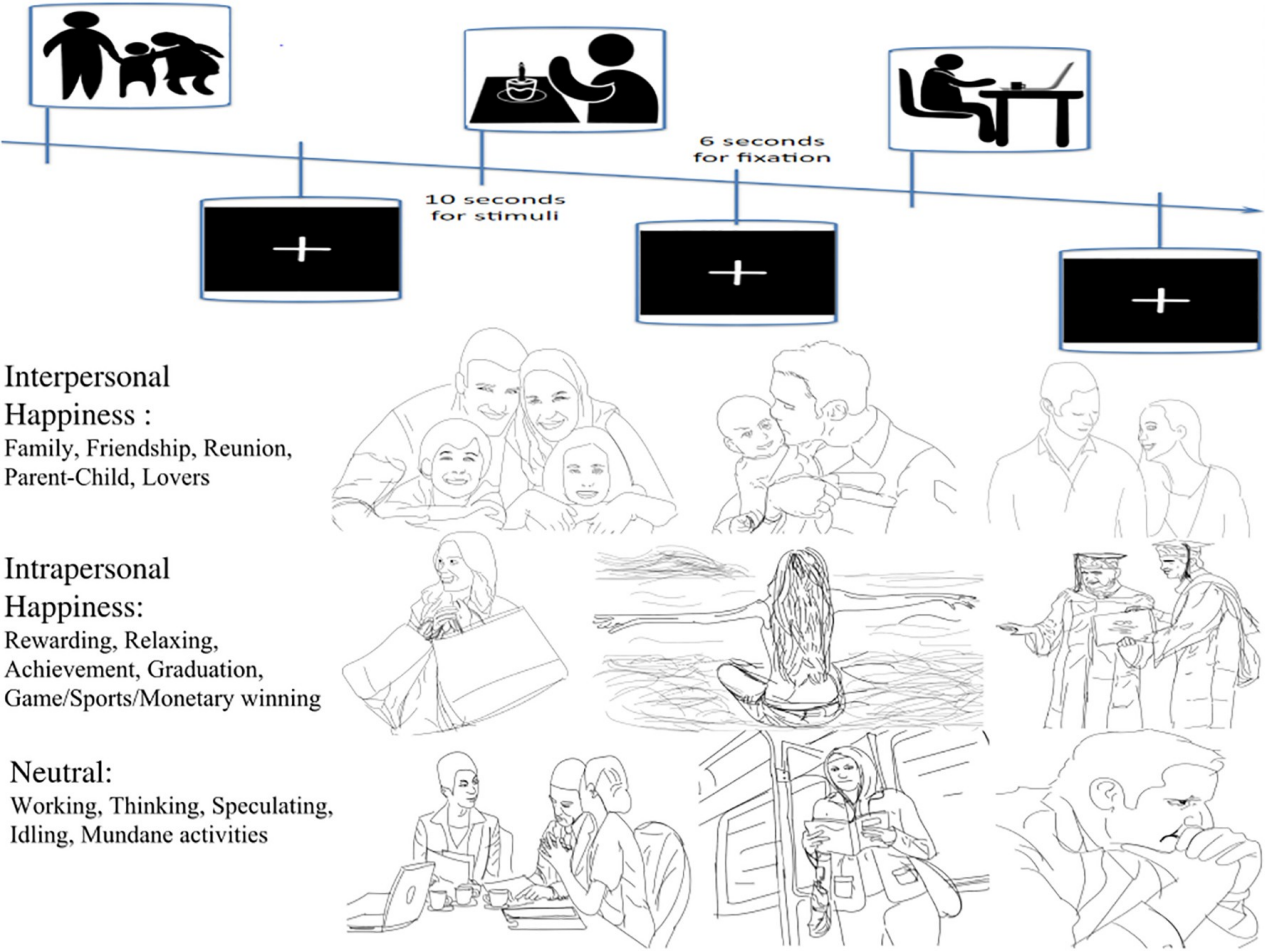
The experimental procedures (top) and examples of stimuli displayed during the experiment (bottom). Three different categories of pictures were googled and rated by a separate group (procedures detailed in the method section): (a) interpersonal/other-concerned wellbeing, including: merry couples, happy family, or company with children, loved ones or significant others); (b) intrapersonal/self-concerned wellbeing, including materialistic reward, tasting delicious food, or winning in sports); and (c) neutral conditions: daily work, mundane moment, walking after work, or idling, etc. To circumvent image copyright issue, the examples shown here were further restricted to include only ‘free for non-commercial reuses’ in Google image search options. The complete set of 550 stimulus pictures of the current study were accessible in public Google Drive (https://drive.google.com/open?id=0B7-b0hGXlfm4Ty03R3R2dWhHT0E).

The functional run consisted of 18 slow event-related trials (6 for each condition), 16s on each trial. Participants were randomly presented with a pictorial stimulus, instructed to engage in the wellbeing state by deeply immersing or projecting oneself in the subjects of picture. Participants’ task was to rate subjective happiness by pressing the corresponding level of button (from 1 to 4). The picture displayed up to 10s (then 6 s of fixation), or was replaced by a fixation immediately after the button press, until 16s trial period expired (Fig 1 bottom). The countdown timing was presented on the lower left side of screen. All the stimuli were presented via MATLAB Psychtoolbox 3.0.10 [36]).

Parallel to our fMRI task instruction (respond by how happy each picture makes subjects feel), a question was raised on the possible conceptual overlaps between intra- vs. inter-personal pictures. To verify, we carried out an online behavioral experiment, where 36 intrapersonal and 36 interpersonal pictures were selected and tested, one at a time, with task instructions being picture-type categorizations (e.g., “if you think the picture belongs to intra-personal type, press ‘a’; or if you think the picture belongs to “interpersonal” type, press ‘h’). Definitions and illustrations of each picture type were the same keyword copies from the stimulus section (and available on the following link: https://www.psytoolkit.org/cgi-bin/psy2.5.3/survey?s=FKCFn&fbclid=IwAR0xK60GZc-A-_nz0-3iuz_u3hp2ESWv6ykB_5AE81_JzrxZE_3kU8aLXjk).

### Image acquisition and data analysis

Images were acquired with a 3T General Electric 750 MRI scanner (GE Medical Systems, Waukesha, WI) at the NCKU MRI center, using a standard 8-channel head coil. Whole-brain functional scans were acquired with a T2*-sensitive EPI (TR = 2 s, TE = 35 ms, flip angle = 76 degree, 40 axial slices, voxel size = 3 x 3 x 3 mm^3^). High-resolution structural scans were acquired using a T1-weighted spoiled grass (SPGR) sequence (TR = 7.5s, TE = 7.7ms, 166 sagittal slices, voxel size = 1 x 1 x 1 mm^3^). Each participant was scanned 5–8 runs, with 5 minutes for each run. The fMRI data were preprocessed and analyzed using BrainVoyagerQX (Brain Innovation, Maastricht, The Netherlands). After slice timing correction, functional images were corrected for head movements using the six-parameter rigid transformations, aligning all functional volumes to the first one in each run. Neither high-pass filtering nor spatial smoothing was applied. The resulting functional data were co-registered to the anatomical scan via initial alignment (IA) and final alignment (FA) and then both fmr and vmr files were transformed into the Talairach space [37].

The volume time courses (VTC) files entered into a general linear model (GLM), including 3 conditions (intrapersonal wellbeing, interpersonal wellbeing, and neutral conditions), each of which was convolved with the canonical (aka. double gamma) hemodynamic response function (HRF). Subsequent linear contrasts (e.g., “intrapersonal wellbeing vs. neutral”) were applied to reveal regions with significant BOLD changes.

Multivariate pattern analyses (MVPA) were performed to complement the univariate GLM approach [38]. MVPA searchlight mapping [35] was applied by first extracting voxel betas for each trial (6) of each condition (3) under a cubic structure (with radius either one or two voxels, extending into 27, or 3x3x3, or 125, or 5x5x5 voxel sets for each voxel), then applying the linear Support Vector Machine (SVM) classifier under Searchmight toolbox [39,40]. The leave-1-trial-out cross-validation was used to test generalization, and the group-level one-sample t-test (against 50% chance level performance) map was done on the combined individual accuracy map.

Furthermore, the parametric modulated GLM was done by coding each trial with both the ‘task’ regressor and the additional parametric regressor (representing the degree of wellbeing button 1–4), so that after the GLM, the main effect of task was separated from the interaction term, the wellbeing-correlated neural responses, presumably linear, with number of subjects (minus one) as the (correct) degrees of freedom. With this manner we were able to identify brain regions that correspond to increases in subjective happiness ratings, and were comparably verified by additional 1–2 vs. 3–4 categorical GLM contrast (see S1 Fig).

To further explore the functional connectivity, Psychophysiological interaction (PPI) analysis [41] was carried out. The seed region NAcc and VTA (TAL xyz coordinates: -10, 8, -6; ~1500 voxels) were defined by searching the term “dopamine” on the meta-analysis based online database, Neurosynth (http://neurosynth.org/). To explore the brain regions functionally connected to seed region more in the interpersonal than in the intrapersonal wellbeing conditions, PPI analyses generated 3 sets of contrasts: psychological (inter- vs. intra-personal), physiological (extracted NAcc/VTA time series under each run), and psychophysiological interactions. The final contrast map was shown as in the PPI result section.

Finally, for the distance-by-condition beta plots (see the hypothesis set 2 section below), the Euclidean distance was calculated from AC point (TAL xyz coordinates: 0, 0, 0) to each voxel of subregions in the pACC/mPFC, and PCC/precuneus. ROIs were defined by the Neurosynth meta-analysis results by searching the above-mentioned brain region.

All the above-mentioned group analyses were conducted in random effects models. And for the visualization of statistical maps, all the statistical tests were set to *p*< 0.05 (cluster threshold corrected), with various uncorrected voxel p-values (p = .05 to p = .000001), each simulated 1000 times (Monte Carlo simulations) and individually estimated smoothing kernel size, by AlphaSim [42] under Neuroelf (V1.1; http://neuroelf.net/). The anatomical labels of peak voxel in significant clusters were determined by Talairach atlas (tdclient), also implemented in the NeuroElf toolbox. The reported coordinates were in the Talairach space.

## Results

### Behavioral results

**Fig 2:**
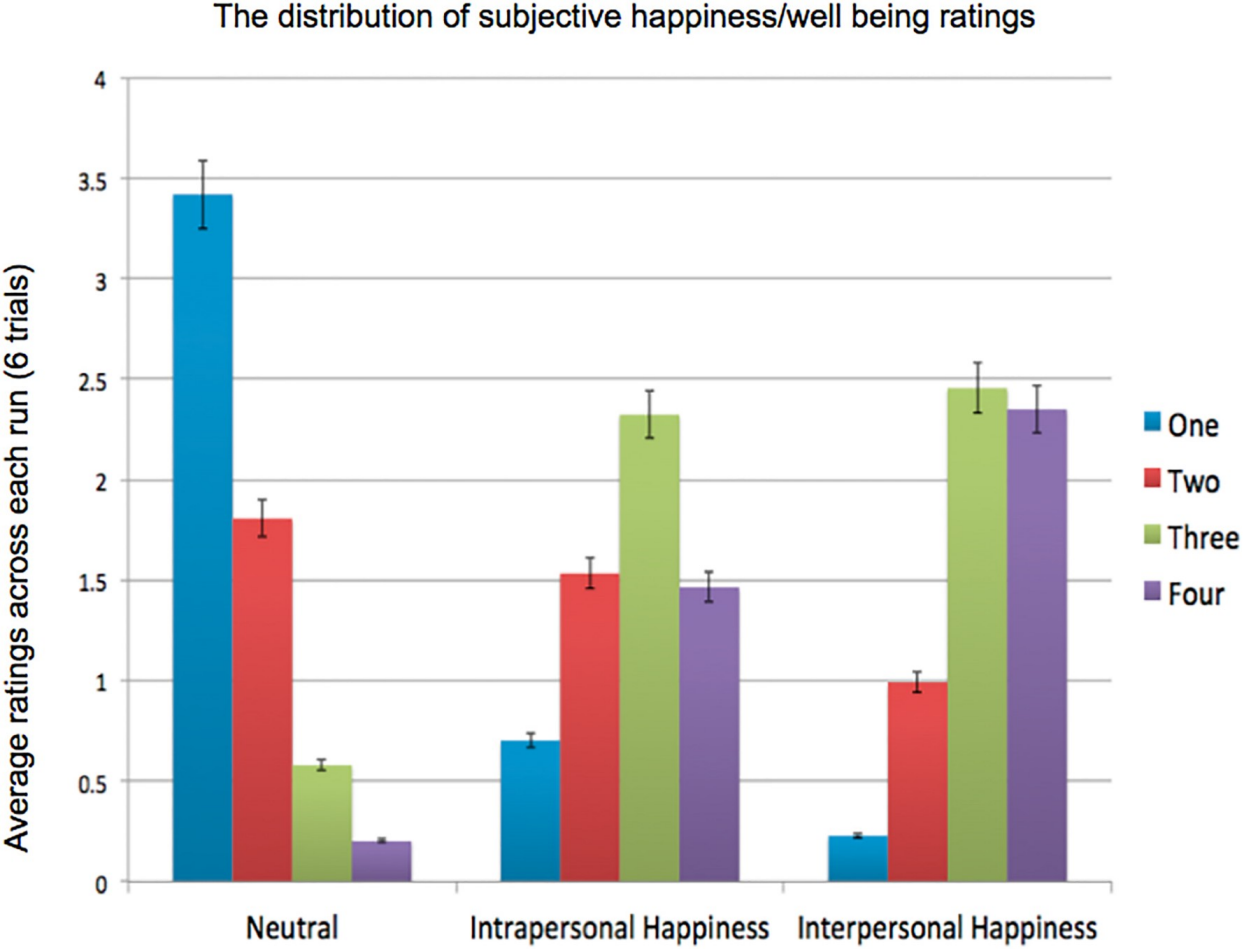
The distribution of subjective happiness/wellbeing ratings for each condition. 1 to 4 denotes the strength of happiness feeling invoked by the individual figure, and emphasized on how each picture make them feel, not evaluating on the people in the pictures. The average happiness ratings were as follows: Interpersonal (3.06) > Intrapersonal (2.69) > Neutral (1.56) conditions.

The bar graphs of mean ratings (1,2,3, and 4) for each trial condition was shown in Fig 2, where participants rated Interpersonal happiness condition as the most happy (Mean = 3.06, SD = 0.42), than that in Intrapersonal happiness condition (Mean = 2.69, SD = 0.41), or Neutral condition, (M = 1.56, SD = 0.34). A one-way ANOVA across 3 trial conditions found its main effect *F*_(2,54)_ = 74.395, *p<*10^−4^, $\eta_{p}^{2}=0.734$; and the ad-hoc multiple comparisons showed significant differences between interpersonal and intrapersonal, between interpersonal and neutral conditions (MeanDiff_inter_vs._intra_ = 0.37, SE = 0.128, *p<*10^−4^, and MD_inter_vs._neutral_ = 1.50, S.E. = 0.128, *p<*10^−4^), and between intrapersonal and neutral conditions as well (MD = 1.13, S.E. = 0.128, *p<*0.01). What is equally important is that the main effect of response times is not significant, *F*_*(2,54)*_ = *0.645, p>0.5*, $\eta_{p}^{2}=0.023$, nor when comparing reaction times across 4 button press conditions, *F*_(2,72)_ = 0.71, *p>0*.*5*, $\eta_{p}^{2}=0.028$. These results suggest that our expected ranking of happiness ratings: “interpersonal > intrapersonal > neutral conditions” was supported, without the joint effect of differential reaction times.

As for the online behavioral experiment, the results suggested that around 1/3 (or 39%) of intrapersonal pictures were categorized as ‘happy/interpersonal’ by all subjects on average, whereas the 36 interpersonal pictures unanimously (94%) categorized as ‘happy/interpersonal’. In other words, while almost all interpersonal pictures were categorized as ‘interpersonal’, some intrapersonal pictures, such as team achievements, share affective and/or conceptual overlaps with the interpersonal component (and therefore be prioritized as ‘happy/interpersonal’). These response asynchrony, while statistically significant (k = 0.558, p < 0.00001), will be shown to be compatible with our fMRI findings (described next), and reflects the need to conduct response-wise (1,2,3, and 4) instead of picture-type-wise (intra- vs. inter-personal), GLM and other analyses.

### fMRI Results

In fMRI, multiple analyses were to test two sets of hypotheses: the first being that, due to the introspective nature of the picture-induced wellbeing task, the central midline structure [43], which hugely overlaps with the default mode network (DMN) [44], and the dopamine reward network [32], which is heavily involved in both pleasure and wellbeing, will both be more activated than from the neutral conditions. Second, according to multiple studies establishing mPFC activation gradients with degrees of pro-sociality [45], or mPFC subdivisions responsive to primary (e.g., food) or secondary (e.g., trinklets) reinforcers [46], we also seek for segregations of such “self- vs. other-concerned” subdivisions in mPFC, and elsewhere (e.g., PCC).

#### Hypothesis set 1: Overlap of wellbeing-related areas and CMS/DMN and dopamine reward circuits

**Fig 3:**
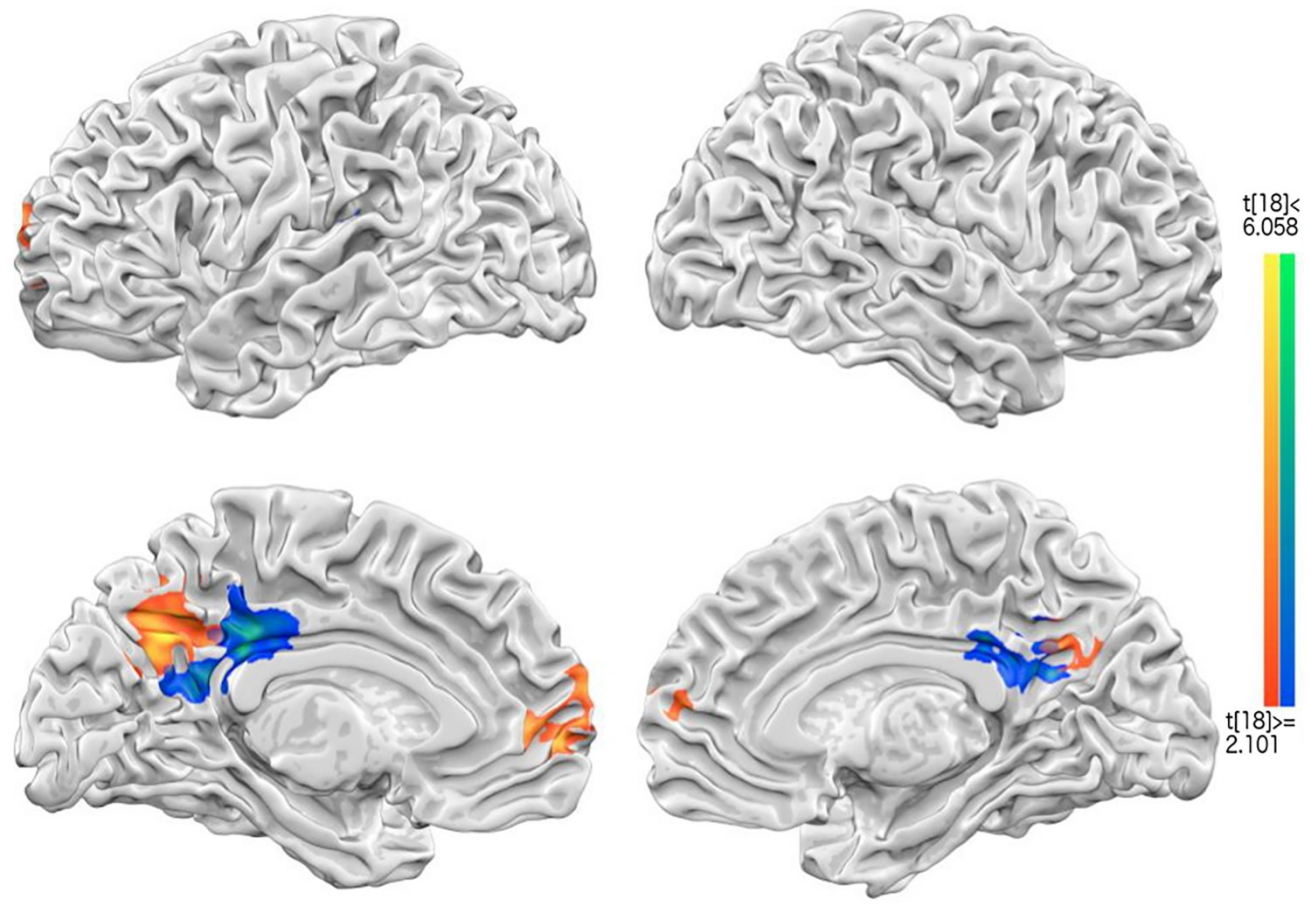
The results of GLM contrast analysis. Interpersonal > Neutral conditions, shown in orange color; and Intrapersonal > Neutral conditions, shown in blue. The medial prefrontal cortex (MPFC) and Precuneus (Prec) were significantly activated for Interpersonal>neutral condition, whereas the posterior cingulate cortex (PCC) were engaged for Intrapersonal > Neutral condition. As can be seen, there was some overlap between the two contrast maps.

To test the first set of hypotheses, two GLMs were separately done for each of the pairwise contrasts for Intrapersonal and Interpersonal, both against Neutral conditions. Fig 3 included the overlay of the two contrasts: “interpersonal > neutral” (orange) and “intrapersonal > neutral” (blue), with a little bit of overlap. The “interpersonal vs. neutral” contrast revealed left dorsal-medial PFC (dmPFC) and bilateral precuneus, and the “intrapersonal > neutral” contrast yielded only the bilateral PCC. The xyz coordinates, cluster size, and t-values of the four GLM contrasts, with the additional “interpersonal vs. intrapersonal”, as well as the “interpersonal plus intrapersonal > 2* neutral” contrasts, are all provided in S1 Table.

Fig 3 revealed that with the categorical GLM contrasts, the commonly activated wellbeing-related regions overlap largely with the Central Midline System (CMS hereafter): mPFC, pACC, and PCC/Precuneus; but not with the expected dopamine reward system: vTA (ventral tegmental area), NAcc (nucleus Accumbens), and putamen, etc. It may be due to the individual differences to each trial, or the diluted BOLD responses sorted by 3 general categories. To get around this limitation by participant idiosyncrasy, additional GLM of the same fMRI data with regressors coded by button presses (1/least-2-3-4/most wellbeing, instead of the pictorial conditions) was carried out, and the contrast of 1–2 vs. 3–4 (see S1 Fig) indeed revealed nucleus Accumbens (nAcc) and nearby brain circuits. Therefore, not only were the results of GLM by picture conditions overlapping with the DMN-/CMS-related brain regions, but the results of GLM by button presses also coinciding the proposed dopamine reward circuits.

**Fig 4:**
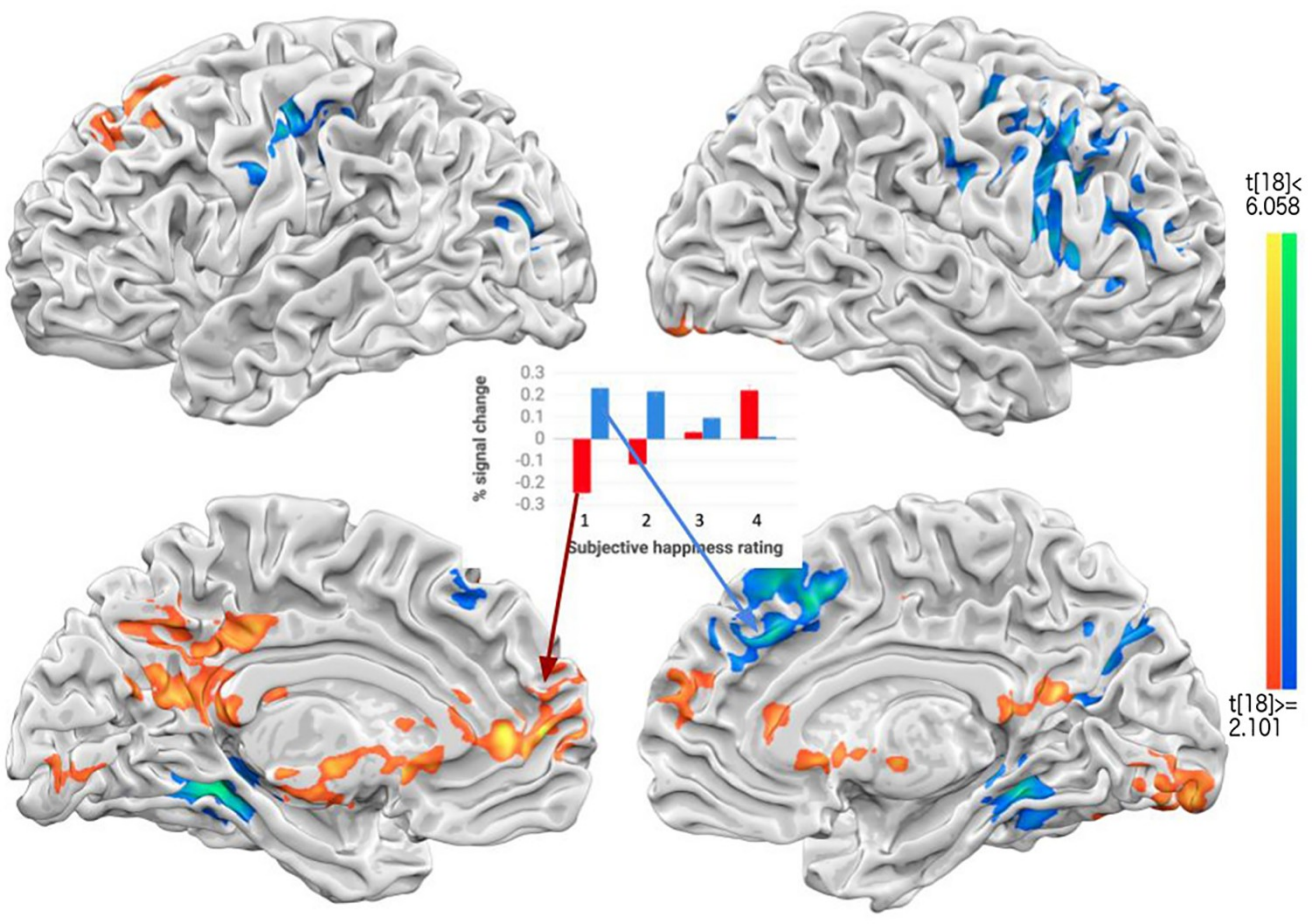
The results of parametric modulation. Medial prefrontal cortex (MPFC), pregenual anterior cingulate cortex (pACC), nucleus accumbens (NAcc), posterior cingulate cortex (PCC), precuneus (Prec), superior temporal gyrus (STG), inferior parietal lobule/TPJ, and insula were positively correlated with subjective happiness ratings, whereas medial frontal gyrus, inferior frontal gyrus, and fusiform gyrus were negatively correlated with the ratings. The central bar graph illustrates two positively and negatively response-correlated brain regions, with their mean percent signal changes (PSC) on each response condition.

To strengthen the GLM results of dopamine reward circuits by categorical contrast with button presses (1, 2 vs. 3, 4) mentioned in S1 Fig, another method of revealing group-level wellbeing-correlated brain regions is parametric modulation (PM) analyses (procedures detailed earlier in method section). The PM results showed that DMN areas, such as precuneus, mPFC, ACC and PCC (all bilateral), right medial insula, and NAcc were highly correlated with increasing levels of subjective wellbeing. Other non-DMN happiness-correlated regions included: the right superior temporal gyrus (rSTG), right inferior parietal lobule (rIPL), bilateral caudate, and bilateral medial temporal gyrus (Fig 4). These PM results provided both additional support and converging evidence that the CMS and reward-related dopamine areas are strongly associated with rated wellbeing, irrespective of types of happiness (e.g., inter- or intra-personal).

**Fig 5:**
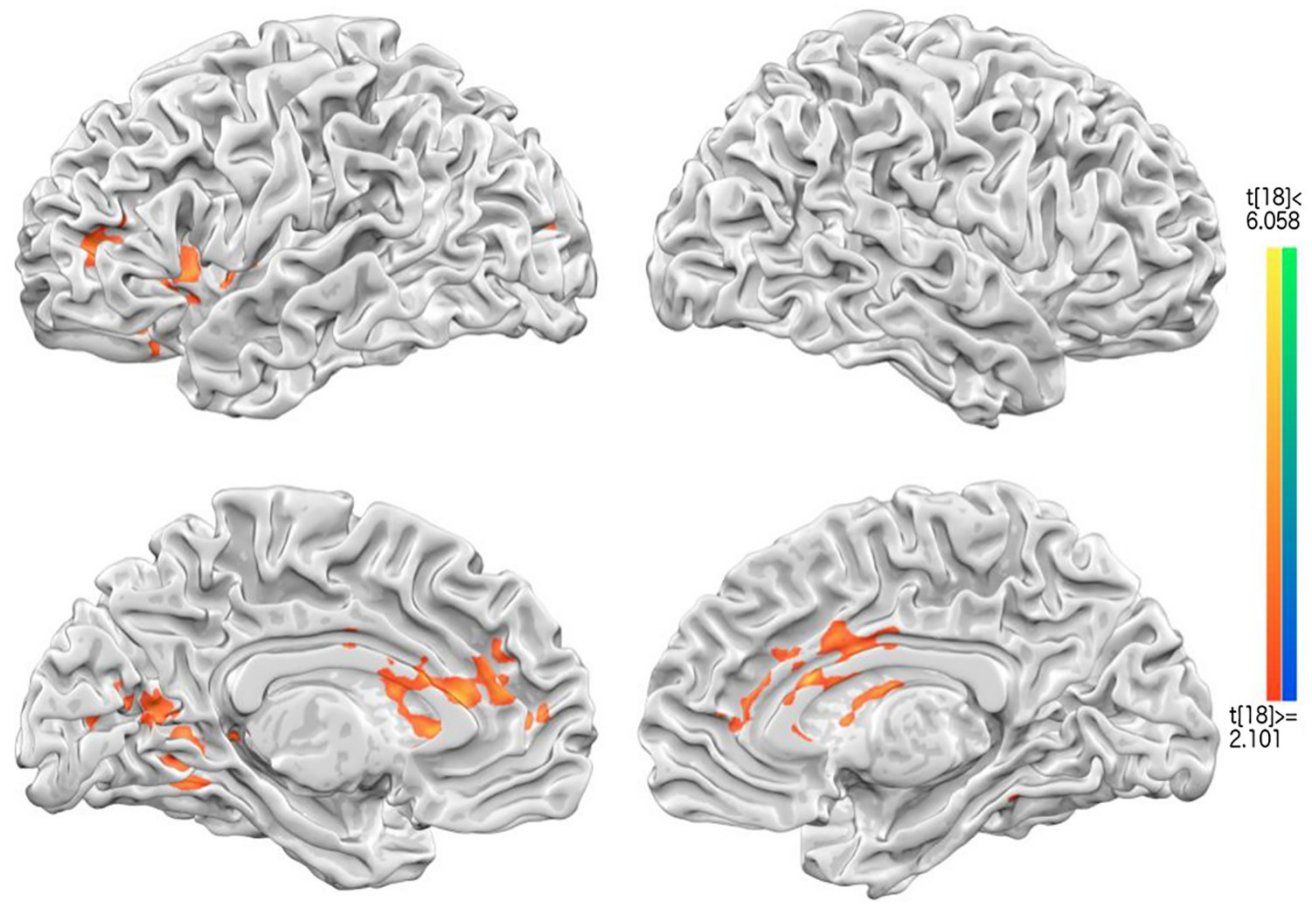
The result of psycho-physiological interaction (PPI). The meta-analysis derived NAcc and VTA (upper panel) were combined to create the seed region. And the functional connectivity analyses were applied. The PPI term revealed the mPFC, Anterior Cingulate Cortex (ACC), and right Insula as the brain regions that are functionally connected with the dopaminergic reward-related seed regions.

In addition to the univariate approach of investigating wellbeing-relation brain regions (GLM and PM), another approach is through functional connectivity, aka. Psychologico-physiological interaction or PPI [47], to see if the two circuits (CMN and reward) also functionally connected to explain wellbeing. To do this, hedonic hotspots: NAcc and Ventral Tegmental Area (VTA) were defined (by typing “dopamine” in neurosynth.org). The functional connectivity map with “interpersonal vs. intrapersonal conditions” revealed mPFC, Anterior Cingulate Cortex (ACC), and right Insula as the brain regions that are functionally connected with the reward seed regions (Fig 5). These findings suggest that the functional coupling between the midline mPFC/rACC and NAcc/VTA area underpins the higher wellbeing more in the interpersonal happiness condition.

**Fig 6:**
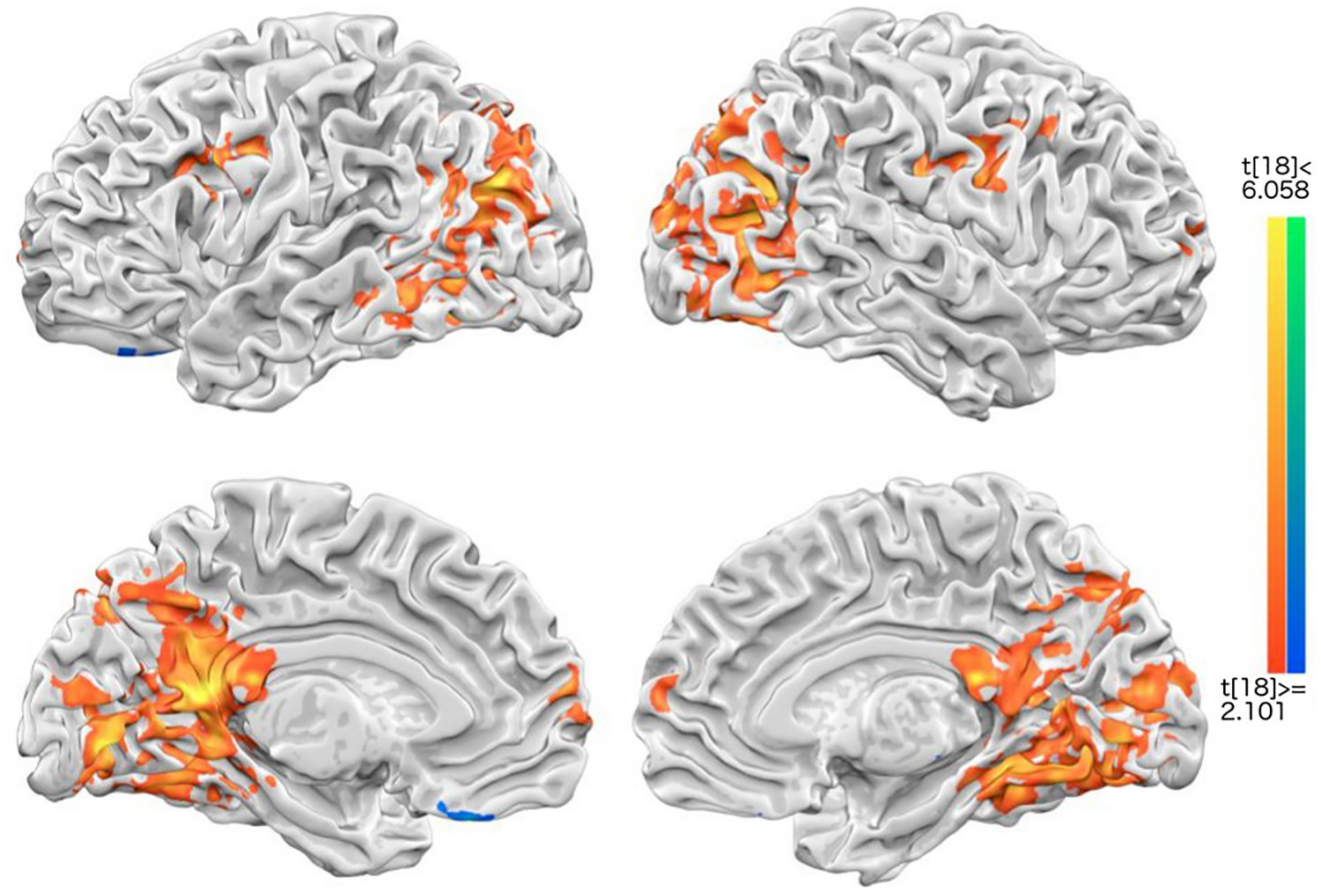
The result of multi-voxel pattern searchlight analysis, with searchlight radius 2 (yielding 5x5x5 = 125 voxels). The brain regions included medial prefrontal gyrus (MPFC), posterior cingulate gyrus (PCC), Precuneus (Prec), precentral gyrus, and superior frontal gyrus, that were significantly above chance (50% classification performance) between Interpersonal and Intrapersonal wellbeing, in a group level.

To complement [38] the abovementioned univariate GLMs, PM, and connectivity (PPI) analyses, multivariate (aka. MVPA searchlight) analysis was also carried out to seek for informational mapping [35]. By the classification performance between intra- and inter-personal wellbeing conditions, the group classification accuracy (with radius 2 only, or 5x5x5 = 125 voxels as a cubic shape) in the dmPFC, PCC/precuneus, middle temporal gyrus, precentral gyrus, and parahippocampal gyrus were significantly above chance (see Fig 6). What is impressive is the high degree of overlap between the univariate (GLM) and multivariate (searchlight) results, especially the bottom row/medial view of both Figs 3 and 4, under the same intra- and inter-personal vs. neutral conditions, providing the convergent findings across the two analysis approaches. Put together, these findings jointly support the expected involvement of CMN and reward circuits in both wellbeing conditions.

#### Hypothesis set 2: Spatial gradients between self- and other-concerned wellbeing

**Fig 7:**
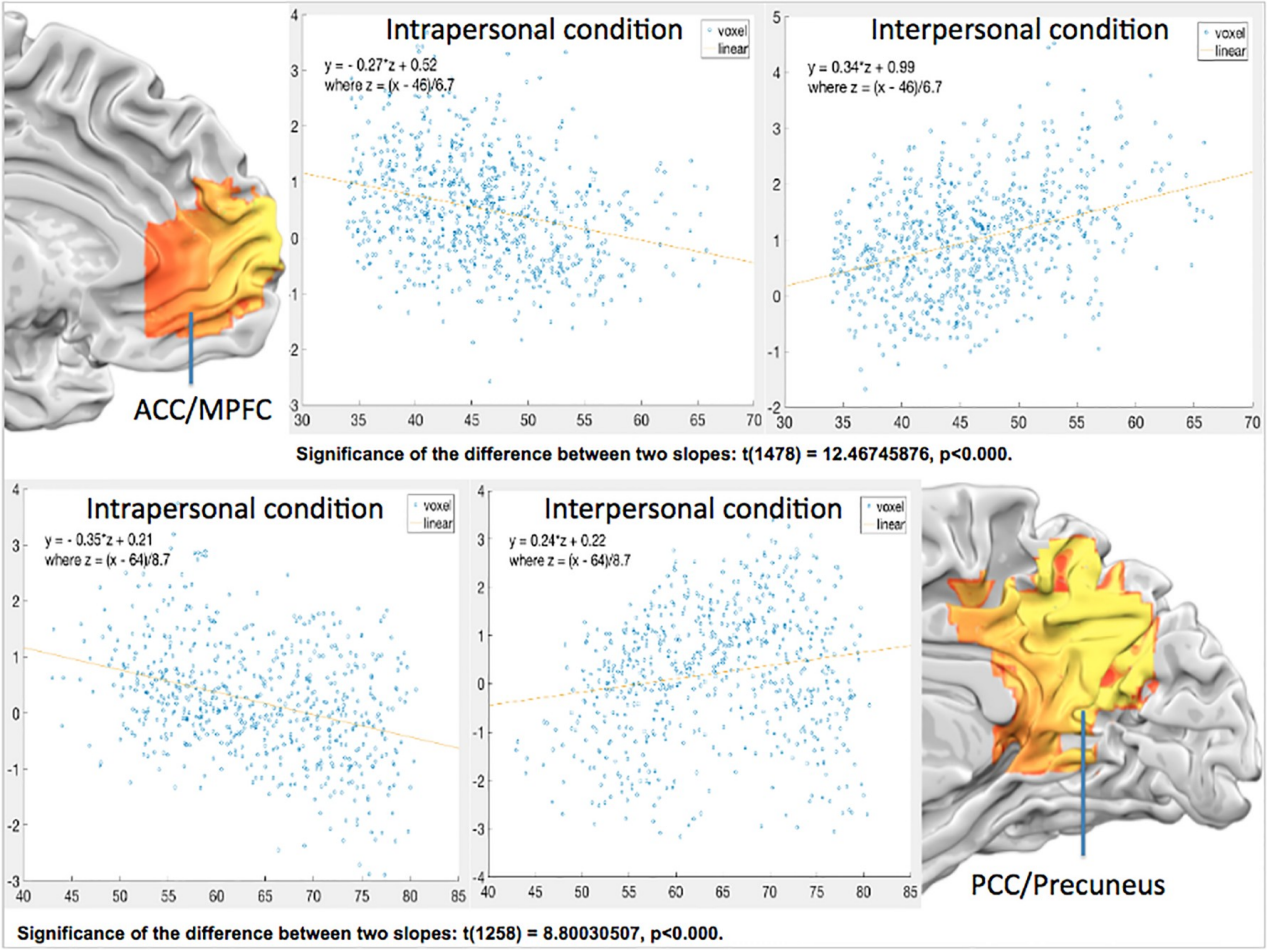
The voxel beta plots of the two core-happiness regions, pACC/MPFC (upper panel) and PCC/precuneus (lower panel), along the rostral-caudal (or y) axis, separately for intrapersonal (left) and for interpersonal (right) wellbeing conditions. The two core regions were delineated via Neurosynth.org, and the numbers for horizontal axis corresponded to y-coordinate value of the activated voxels. The regression lines and equations were provided for visualization and comparison purpose: the voxels in both regions showed decreasing betas in intrapersonal, and increasing betas for interpersonal wellbeing, with increasing distance with AC (Anterior Commissure) point.

In addition to our first set of hypothesis (that two sets of areas would be more activated in both wellbeing conditions), the 2^nd^ prediction of spatial arrangements of intra- vs. inter-personal wellbeing has been tested here along the cortical midline brain areas. The maps of relative contrast of intra- or inter-personal vs. neutral condition (Fig 3) lend initial support for the segregation of intra- vs. inter-personal wellbeing in both mPFC (interpersonal vs. neutral) and precuneus/PCC (both intra- and inter-personal vs. neutral). Fig 3 (groupwise GLM) and 6 (groupwise searchlight) further reinforce this distance gradient with both univariate and multivariate analyses, showing that only the outer/anterior mPFC and posterior PCC regions were recruited by “interpersonal vs. neutral” in Fig 3, and “inter- vs. intra-personal” contrast in Fig 6. Last and most importantly, to quantitatively compare the differences between the voxels underpinning intra- and interpersonal wellbeing conditions, we first extracted mPFC and PCC voxels from neurosynth.org (overlaid in Fig 7), and then calculated their Euclidean distances of each voxel to Anterior Commissure (AC, Talairach coordinates: 0, 0, 0) as common reference. In general, pACC/mPFC voxels were located 35 to 55 voxels from AC, whereas PCC/precuneus 50 to 80 voxel distances. The beta scatterplots for intrapersonal (blue) and interpersonal (orange) wellbeing, along with their linear regression lines, were overlaid together next to their anatomical locations. For the intrapersonal condition, mPFC voxels showed decreasing activations with further distance from AC (blue), and increasing activations (orange) with further distance from AC in the interpersonal condition (Fig 7 upper panel). Likewise, PCC voxels also showed this “decreasing activations with further distance from AC in intrapersonal; increasing activations with distance in inter-personal condition” trend, commonly reflecting their separate activation profiles in two different conditions. The slope of intra-/inter-personal regression lines were significantly different in both brain areas (*t*(1478) = 12.467, *p<*10^−4^) in ACC/MPFC; *t*(1258) = 8.8, *p<*10^−4^ in PCC/Precuneus). To summarize the findings in hypothesis 2 set, not only were our findings of “increasing for inter-, decreasing for intra-personal” mPFC activations consistent with previous literature bearing the similar topic (but with different tasks and analysis methods, [45,46], but extending from the previous focus of mPFC to including PCC as well, making our find a broader coverage of the central midline circuit.

## Discussions

The current study began with the review of the philosophical backgrounds and historical progressions of the wellbeing concept, including the distinction of hedonic and eudemonic wellbeing, emotional vs. cognitive aspects of happiness, to the latest of most relevant self- vs. other-concerned wellbeing. After 90s, with the explosion of neuroimaging (especially fMRI) approaches and its widespread coverage of topics, the neural substrate of subject wellbeing or happiness was surprisingly scarcely explored: from the memoir moment of past achievements by Hollywood movie stars [29], to the unexpected momentary gain in the gaming context [30] as their separate operational definition of happiness/wellbeing, the implicit acknowledgement was its elusive nature, and the flexibility (or difficulty) to create happiness manipulations suitable under the constraints of fMRI settings. Considering these, the present study adopted one of the most recent perspectives of wellbeing: self- vs. other-concerned [21,48], designed the slow event-related fMRI experiments, where participants judged each picture, independently categorized as “interpersonal wellbeing”, “intrapersonal wellbeing”, or “neutral” conditions. In short, the present design not only addressed the dual idiosyncrasies of individual differences to wellbeing and to the meaning of different pictures to each individual, but also balanced between the incubation and flow of wellbeing, and between the trial numbers (the more the better) and trial lengths (longer is better, but not too long). According to our main behavioral results, most participants responded within 3 seconds, and no systematic RT differences among 3 stimulus categories (nor for 4 response categories), reflecting that most participants used a safe strategy of responding as soon as they could tell their mental state (e.g., degree of happiness), and did not dwell in the “sucked-in, timeless” state for too long (our naïve expectation was that at least in some trials, the RT will be sometimes longer than the 10s trial countdown limit). Although nearly 40% of the intra-personal pictures were categorized as interpersonal trials (whereas 94% of interpersonal pictures were categorized as interpersonally), by most subjects of a separate behavioral experiment, it just suggests the conceptual/affective overlap between intra- and inter-personal conditions, and does not invalidate our fMRI task (e.g., both design and instructions were different).

To recap, our fMRI results generally were in line with two sets of predictions: first, happiness or wellbeing manipulations generally induced central midline structures: MPFC, Ventral striatum/nucleus accumpens, PCC/Precuneus (reference of positive affect or traditional happiness neural substrate; meta analysis results [49]), along with other associated brain areas, such as superior temporal gyrus [50], insula [27], and TPJ [51,52] by GLM contrast (Fig 3) or by the response-related correlations (via parametric modulation analyses, Fig 4). These may not be surprising given that the literature has shown the involvement of CMS (including ACC/MPFC and PCC/Precuneus), default mode networks (above mentioned areas plus the IPL), and reward circuit (VTA/NAcc) in wellbeing [25,31,53–55]. Besides, our results are also in agreement with the expectation that higher other-concerned wellbeing should induce higher activities from theory-of-mind (TOM) areas [56]. What is more, PPI analysis (Fig 5) further revealed that interpersonal wellbeing, whose ratings were generally higher than those on intrapersonal ones, also showed higher functional connectivity between dopaminergic VTA/NAcc areas and ACC/MPFC, together as the “hedonic hotspot” [32], furthering their importance underlying participants’ SWB.

The second sets of hypothesis mainly concern the neuronal mapping of self- vs. other-concerned wellbeing in the brain: that self-concerned/intrapersonal SWB involved more inner CMS, and other-concerned/interpersonal SWB outer CMS (mainly ACC/MPFC and PCC/Precuneus). These hypotheses received two ways of support from both GLM contrasts (“intrapersonal vs. neutral” and “interpersonal vs. neutral” conditions) and MVPA searchlight analyses: the former (GLM, Fig 3) showed close but separate CMS regions (with intrapersonal wellbeing more in PCC, and interpersonal wellbeing anterior MPFC and Precuneus), and the latter (MVPA, Fig 6) showed anterior MPFC and a broad PCC/Precuneus were both significantly above-chance in classifying between intra- vs. inter-personal wellbeing conditions.

Our 2^nd^ set of evidence, characterized as “inner to outer CMS to self- to other-concerned gradients” mapping, can be emphasized or reinterpreted in Fig 7: by calculating the Euclidian distances to the common AC point (xyz: 0, 0, 0 in Talairach space), the activated voxels in both intra- and inter-personal conditions that fell between the pACC/MPFC and PCC/Precuneus regions, were plotted with their regression line (equation written in the upper left of each subplot). While the intra-personal wellbeing showed decreasing slope, and the interpersonal wellbeing showed increasing slope along the y axis in ACC/MPFC; the trend was reversed in the case of PCC/Precuneus region (Fig 7). In the case of ACC/MPFC, our results were in line with earlier studies that suggested the similar self-to-other dimensions along the medial MPFC and orbitofrontal cortex: including the concrete to abstract hedonic gradient [57], primary (e.g., food) to secondary (e.g., trinklets) reinforcers [46], to the money donation allocated (altruistic processing) [45]. What is new in the current study, and also the first to our knowledge, is the similar findings in the PCC/Precuneus. As summarized, the two core CMS both showed the self-to-other-focused distribution along the y axis, with ACC/MPFC and PCC/Precuneus showing reverse directions. These results echo a recent cross-species (humans vs. Macaques) comparative resting-state fMRI study [58], that provided the anatomical account of why the DMN is the ideal origin of human cognition: because of its equal (geodesic) distance to all external (including visual, auditory, and sensorimotor) processing areas. With increasing abstract representation processed by multimodal areas, the extreme of the processing hierarchy, DMN, is the ultimate site for the most abstract ‘self’ processing. Our findings of the self-to-other gradients in pACC/MPFC and PCC/Precuneus, two of the important DMN hubs, are consistent with this “processing gradient principle”, and therefore mutually supportive for each.

One limitation of the current findings was the lack of participants from various age groups, especially the older populations. However, Siedlecki, Salthouse, Oishi, & Jeswani [16] examined various kinds of subjective well-being (N = 1111), and found no substantial differences across young, middle-aged, and older participants in various indices of wellbeing: enacted and perceived social support, family embeddedness, and provided support. That said, our naïve expectations about different response profiles for intra- and interpersonal wellbeing trials between young and old age groups have not been examined. Future studies would still be needed to verify the generality of our current findings, mainly by recording youngsters’ brain, to older age groups.

Lastly, two CMS regions, MPFC/pACC and PCC/Precuneus heavily implicated in our intra- and inter-personal wellbeing conditions, could be either viewed as one bigger circle of the encompassing self, or two smaller circles around only MPFC and PCC regions (but still follows the layered self-to-other structure). Although only the latter version of these two speculations got some extra support [58], both could be viewed as the adult self with growing awareness from self concentration, to concerning for close or significant others, and then gradually for the bigger self, even to the biospheric concept of a citizen of the planet earth [59]. Future studies could also elaborate on these possible mappings and shed more light on the intricate interrelations among the dynamic self, their inner brain, and the person-environment interactions.

## Conclusion

By demonstrating (i) engagement of CMS and related brain areas in response to the affective and cognitive states induced by intra- and inter-personal wellbeing; and (ii) higher connectivity between MPFC and dopamine reward circuit and higher wellbeing, and (iii) overlapping MPFC/pACC and distinctive PCC/Precuneus activities/patterns for intra- and inter-personal wellbeing representation, the current findings provide strong neural evidence for the collaboration of various self- and reward- reward brain areas underpinning the social wellbeing.

## Supporting information

S1 Table GLM contrast table of significant cluster(s) in 4 different contrasts. (DOCX)

S1 Fig The GLM with regressors by subjective ratings of happiness (1, 2) vs. (3, 4). Notice that in addition to the high similarity between the activated/deactivated areas in S1 Fig and Fig 4, the central illustrative bar graph also differed in how the conditions were coded: (1, 2) vs. (3, 4) in the present figure, vs. (1,2,3, and 4) with linearity assumed among regressors.
(DOCX)
