# Supplementary material for: The neural substrate of self- and other-concerned wellbeing: An fMRI study
Source: PLoS One. 2019 Oct 1;14(10):e0203974. doi: 10.1371/journal.pone.0203974 (PMC6772049; doi:10.1371/journal.pone.0203974)
Supplement: S1 Table — (DOCX) [file pone.0203974.s001.docx]

**S1 Table. GLM contrast table of significant cluster(s) in 4 different contrasts**

Type of map: t-Map

Degrees of freedom: 18

Cluster k-threshold: 3456 mm^3 (128 voxel)

Applied map threshold: 2.10092 (p < 0.05000)

Type of coordinates: peak

Interpersonal > neutral

x y z | k | max | mean | tdclient

-----------------------------------------------------------

-3 -64 31 | 172 | 4.932078 | 2.806057 |LH Cuneus (Brodmann area 7)

-4 54 -1 | 140 | 4.267472 | 2.605303 |LH Anterior Cingulate (Brodmann area 10)

27 -52 -9 | 637 | -7.627149 | -2.998649 |RH Fusiform Gyrus (Brodmann area 19) ( 26; -52; -9) [d=1.0mm]

48 14 26 | 261 | -6.649028 | -2.824088 |RH Middle Frontal Gyrus (Brodmann area 9)

-30 -42 -3 | 721 | -6.505451 | -2.871893 |LH Parahippocampal Gyrus (Brodmann area 19)

-32 44 39 | 137 | -4.961943 | -2.665316 |LH Middle Frontal Gyrus (Brodmann area 9) (-32; 44; 37) [d=2.0mm]

-57 27 3 | 128 | -3.483229 | -2.474208 |LH Inferior Frontal Gyrus (Brodmann area 45)

Intrapersonal > neutral

x y z | k | max | mean | tdclient

-----------------------------------------------------------

-6 -50 18 | 330 | 4.401576 | 2.612963 |LH Posterior Cingulate (Brodmann area 30) ( -6; -51; 17) [d=1.4mm]

Interpersonal + Intrapersonal > 2*neutral

x y z | k | max | mean | tdclient

-----------------------------------------------------------

-19 -21 32 | 134 | 4.654984 | 2.627876 |LH Cingulate Gyrus (Brodmann area 31) (-18; -21; 38) [d=6.1mm]

-6 -66 33 | 252 | 4.136971 | 2.665458 |LH Cuneus (Brodmann area 7)

-30 -45 -6 | 218 | -6.002037 | -2.789952 |LH Parahippocampal Gyrus (Brodmann area 37) (-30; -45; -7) [d=1.0mm]

30 -38 -3 | 126 | -5.054093 | -2.963882 |RH Sub-Gyral (Hippocampus) ( 30; -37; -2) [d=1.4mm]

48 14 26 | 274 | -4.989686 | -2.760589 |RH Middle Frontal Gyrus (Brodmann area 9)

Interpersonal > Intrapersonal

x y z | k | max | mean | tdclient

-----------------------------------------------------------

27 -52 -9 | 829 | -6.540304 | -2.793211 |RH Fusiform Gyrus (Brodmann area 19) ( 26; -52; -9) [d=1.0mm]

-27 -36 -5 | 395 | -4.550956 | -2.704958 |LH Parahippocampal Gyrus (Hippocampus) (-28; -35; -5) [d=1.4mm]

-34 -75 23 | 178 | -4.486015 | -2.693272 |LH Superior Occipital Gyrus (Brodmann area 19) (-35; -75; 26) [d=3.2mm]
