# Supplementary material for: The neural substrate of self- and other-concerned wellbeing: An fMRI study
Source: PLoS One. 2019 Oct 1;14(10):e0203974. doi: 10.1371/journal.pone.0203974 (PMC6772049; doi:10.1371/journal.pone.0203974)
Supplement: S1 Fig — Notice that in addition to the high similarity between the activated/deactivated areas in S1 Fig and Fig 4, the central illustrative bar graph also differed in how the conditions were coded: (1, 2) vs. (3, 4) in the present figure, vs. (1,2,3, and 4) with linearity assumed among regressors. (DOCX) [file pone.0203974.s002.docx]

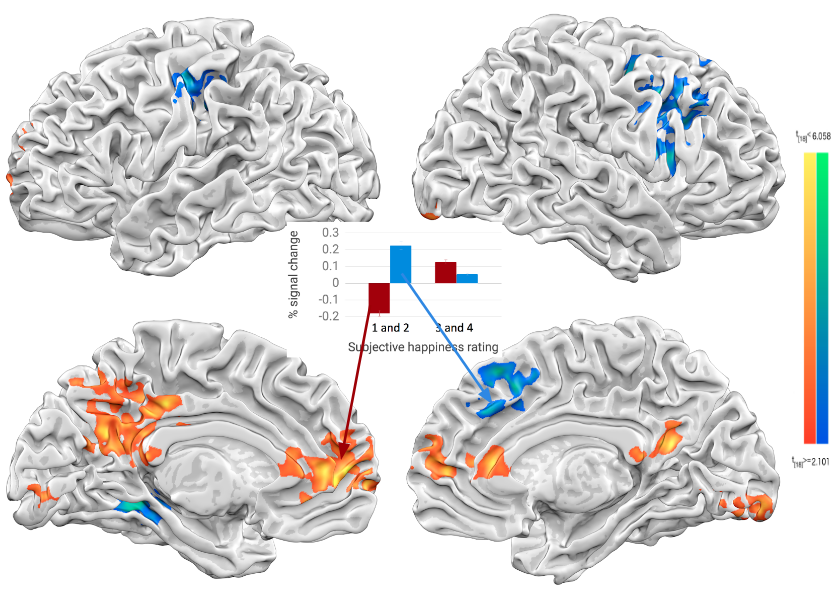


**S1 Fig. The GLM with regressors by subjective ratings of happiness, (1, 2) vs. (3, 4).** Notice that in addition to the high similarity between the activated/deactivated areas in S1 Fig and Fig 4, the central illustrative bar graph also differed in how the conditions were coded: (1, 2) vs. (3, 4) in the present figure, vs. (1,2,3, and 4) with linearity assumed among regressors .
